# Cost Effectiveness of a Shorter Moxifloxacin Based Regimen for Treating Drug Sensitive Tuberculosis in India

**DOI:** 10.3390/tropicalmed7100288

**Published:** 2022-10-08

**Authors:** Malaisamy Muniyandi, Nagarajan Karikalan, Banurekha Velayutham, Kavitha Rajsekar, Chandrasekaran Padmapriyadarsini

**Affiliations:** 1 ICMR—National Institute for Research in Tuberculosis, Chennai 600031, India; 2 Department of Health Research, Ministry of Health and Family Welfare, New Delhi 110001, India

**Keywords:** tuberculosis, short course TB regimen, cost-utility, life years gained, QALYs gained, shorter moxifloxacin-based regimen

## Abstract

Globally efforts are underway to shorten the existing 6-month tuberculosis (TB) treatment regimen for drug-sensitive patients, which would be equally effective and safe. At present, there is a lack of evidence on the cost implications of a shorter 4-month TB regimen in India. This economic modeling study was conducted in the Indian context with a high TB burden. We used a hybrid economic model comprising of a decision tree and Markov analysis. The study estimated the incremental costs, life years (LYs), and quality-adjusted life years (QALYs) gained by the introduction of a Moxifloxacin-based shorter 4-month treatment regimen for pulmonary TB patients. The outcomes are expressed in incremental cost-effectiveness ratios (ICERs) per QALYs gained. The cost per case to be treated under the 4-month regimen was USD 145.94 whereas for the 6-month regimen it was USD 150.39. A shorter 4-month TB regimen was cost-saving with USD 4.62 per LY and USD 5.29 per QALY. One-way sensitivity analysis revealed that the cost of the drugs for the 4-month regimen, hospitalization cost for adverse drug reactions, and human resources incurred for the 6-month regimen had a higher influence on the ICER. The probability sensitivity analysis highlighted that the joint incremental cost and effectiveness using QALY were less costly and more effective for 67% of the iteration values. The cost-effectiveness acceptability curve highlights that the 4-month regimen was dominant to both patients and the National TB Elimination Programme in India as compared to the 6-month regimen at different cost-effectiveness threshold values.

## 1. Introduction

Studies on the efficacy of a 4-month treatment regimen for drug-sensitive tuberculosis (TB) have been evaluated in many countries [1,2]. Shortening TB treatment duration is expected to reduce the health system and patient burden in terms of time and resources spent [3]. A systematic review of the clinical efficacy of shortened TB treatment regimens highlighted their non-inferiority to the standard 6-month regimen [2]. At present, multiple trials are underway to test the clinical efficacy of shortened TB treatment regimen and few have been published. With the available evidence, there is optimism toward effectively reducing the treatment duration of TB at the population level [4]. While clinical efficacy findings of shortened regimen are increasingly available, still there is a lacuna concerning the cost implications of shortened regimens.

India with an annual incidence of 2.6 million TB cases is striving to accelerate the incorporation of evidence-based new interventions in its National TB Elimination Programme (NTEP) to achieve the TB elimination goal by 2025. Since 2017 several new initiatives have been undertaken to control TB more efficiently and shortening of the TB treatment duration is considered an important strategy to achieve the TB Elimination Goals [5]. 

Cost-effectiveness model-based analysis using primary data from Bangladesh, South Africa, Brazil, and Tanzania showed that the 4-month regimen was non-inferior and cost-effective in Bangladesh and cost-saving in other settings [6]. A similar study in South Africa demonstrated that a 4-month regimen would be cost-saving for patients and cost-effective for the health system at USD 436 per month at the willingness to pay (WTP) threshold of one Gross Domestic Product (GDP) per capita [7]. A population-based modeling study estimated that the implementation of shortened 4-month regimen could reduce TB mortality by 3.5% over ten years period compared to a 6-month regimen of equal efficacy [8]. Currently, under NTEP the globally recommended 6-month regimen for drug-sensitive pulmonary TB is being administered [9]. While the effectiveness of this regimen has been established still the rate of adherence, and lost-to-follow-up are considerable, which has a profound impact on the clinical outcomes and economic impacts [10]. To address this, shortening the duration of the TB treatment regimen is considered a priority for research and implementation under NTEP. Recent evidence on the clinical efficacy of a Moxifloxacin-based shortened regimen was found to be efficacious as the 6-month regimen [11]. Further multicentric studies are in pipeline to estimate the population-level effectiveness of the shortened regimens in India. While this is important progress towards achieving shorter and more effective treatment interventions for TB in India, still the economic aspects in this regard remain unanswered. The economic implications of shortened regimen require a priority research focus equal to its clinical implications. Understanding the cost-effectiveness of shortened regimens from a provider perspective could inform the long-term cost implications for NTEP in India. In addition, this could also inform the cost implication for TB patients experiencing catastrophic expenditure. In this background, we have attempted to assess the cost-effectiveness of a Moxifloxacin-based 4-month TB regimen.

## 2. Materials and Methods

We used a hybrid economic model involving a decision tree and Markov modeling from a societal perspective for this economic evaluation. In the current modeling work, we focused on assessing the impact of the current standard 6-month TB treatment regimen and a shortened 4-month regimen based on a hypothetical cohort of TB patients undergoing treatment in the public health facilities of a high TB burden country. 

### 2.1. Intervention and Comparator

The current 6-month regimen for drug-sensitive TB consists of Isoniazid, Rifampicin, Pyrazinamide, and Ethambutol for the initial 2-month intensive phase followed by Isoniazid, Rifampicin, and Ethambutol for the subsequent 4-month continuation phase (2HRZE_7_/4RHE_7_) [12]. The model compares this regimen with the 4-month regimen consisting of Moxifloxacin, Isoniazid, Rifampicin, Pyrazinamide, and Ethambutol for the initial 2 months followed by Moxifloxacin, Isoniazid, and Rifampicin for the subsequent 2-months (2RHZEM_7_/2RHM_7_) (Table S1). The proposed 4-month regimen [11] was found to be non-inferior to the standard 6-month regimen, based on the TB treatment outcomes such as a cure rate of 92% vs. 93%, a failure rate of 2% vs. 1%, a lost-to-follow-up rate of 4% vs. 4% and a death rate of 3% vs. 4%. The current analysis aims to study the impact of treatment shortening alone. We did not include non-inferiority margins and instead set the two regimens to be almost equivalent in their immediate clinical outcomes. However, the difference between regimens in terms of adverse drug reaction was 6% [11] vs. 13% [13,14] and recurrence was 4% vs. 6% in 4-month and standard 6-month regimens, respectively. 

### 2.2. Time Horizon

A lifetime horizon is considered to model the cost and outcomes of the two compared regimens. From the literature, we found that the average age of TB patients was 32 years and thus the life expectancy at age 32 of 44 years was used. TB treatment regimen remains the same for all age groups except children. Our model considered only adult patients. With this average age of the cohort, life expectancy and all-cause mortality were calculated using the standard life table of India. A global annual discount rate of 3% was incorporated for both the cost and consequences [15]. This model characterized the health state of the cohort and it was followed until cure or death. 

### 2.3. Model Description

The model followed up a standard hypothetical cohort of 100,000 drug-sensitive TB patients with an average age of 32 years [16]. We considered only patients who accessed the public health facilities fortnightly for medication. The model considered treatment cure, lost-to-follow-up, failure, and death as the clinical outcomes (Table S2) between the two regimens. Demographic charecteristics of the TB patient from different studies are given in Table S3. The model outcomes are life years (LYs) gained and quality-adjusted life years (QALYs) gained by patients treated in two different regimens. Disease recurrence and retreatment were considered transition health states. Adverse drug reaction (ADR) and drug resistance attributable to the treatment regimen were also considered (Supplementary File S1).

### 2.4. Decision Tree

The decision tree planned for this study was constructed based on the treatment cascade of both 4-month and 6-month regimen and probabilities associated with different health states and outcomes (Figure S1). Both the proposed 4-month regimen and the current strategy of the 6-month regimen were modeled as two parallel trees using probabilities associated with the treatment outcomes. A patient put on TB treatment in each strategy was further classified based on the adverse drug effects and no adverse drug effects. Microsoft Excel spreadsheet and TreeAge Pro 2020 (TreeAge Software Inc., Williamstown, MA, USA, Licensed version 2020 R 1.0) was used for analysis.

### 2.5. Markov Model

**Figure 1:**
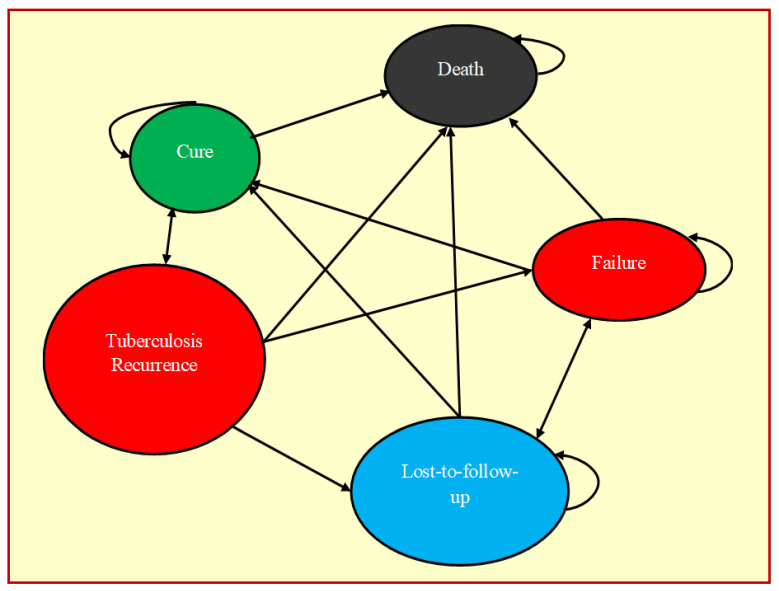
Markov model of the sequence of possible TB treatment outcomes and health states.

A total of five health states were considered for the Markov model which included cure, failure, loss-to-follow-up, death, and TB recurrence. Each patient spent a one-year cycle in the model and further they moved into another health state based on the transition probabilities. Markov model was used to assess the transfer of individuals between different health states. Drug-sensitive TB patients who are on treatment could be cured, and the cured individuals may transition to death or recurrence. Treatment lost-to-follow-up patients may move to cured, death, or failure states. Treatment failure patients may move to death, cure or lost-to-follow-up. Death due to TB was the absorption state from which no transition occurred. We used one-time probability for treatment outcomes of drug-sensitive TB and annual probability for drug-resistant TB since the recurrence occurred mostly within a year. The transition process between the health states is provided in Figure 1 and Figure S2.

### 2.6. Model Input Parameters

The key input parameters for the model included age-specific life expectancy and all-cause mortality [17]. The cohort starts with an average age of 32 years and the life expectancy at age 32 years was considered as 44 years. TB treatment regimen remains the same for all age groups except children. Our model considered only adult TB patients. With this average age of the cohort, life expectancy and all-cause mortality were calculated using the standard life table of India. The other input parameters are the probability of cure, lost-to-follow-up, failure, death due to TB, recurrence of TB, retreatment, and ADR [11,12,13]. The clinical outcomes for the 4-month regimen were collected from the randomized controlled clinical trial evidence on the efficacy of a Moxifloxacin based shortened regimen in India, and the clinical outcomes for the 6-month regimen were collected from the NTEP reports of India and from published multi-centric study evidence. Currently, in the NTEP, all notified drug-sensitive TB patients are treated with 6-month treatment and our proposed intervention is a 4-month regimen. 

Cost Data: This cost-effectiveness modeling was conducted primarily from a societal perspective which included costs incurred by the NTEP (health system cost) and the costs (patient cost) incurred by the individual who accesses treatment services for TB. The costs for both treatment regimens such as medication [18], an investigation [19], human resources used [20], hospitalization for ADR [21], and drug resistance [22] were considered as health system costs. The medication costs were collected from the National Pharmaceutical Pricing Authority which is supplying drugs. Human resource costs included the proportion of time spent on TB services by different health personnel ranging from 100% for personnel working in the district TB center to <10% for health visitors working in the field. This was taken from the previous study conducted at our center. 

Despite the free treatment services provided under public health facilities in India, patients incurred considerable out-of-pocket expenditures for their treatment. Therefore, the societal perspective was considered to account for the cost incurred by TB patients for availing treatment services in public health facilities in the form of direct and indirect non-medical costs. The costs for food [23], travel [24], and attendees [25] during treatment were considered direct non-medical costs and loss of income [26] was considered indirect non-medical costs. All these costs were collected from different independent studies conducted in India. Further, all these costs were calculated for the full episode of treatment in both regimens, which have different durations. The costs were estimated in Indian rupees and further converted to USD (USD 1 = INR 74.35) based on the exchange rate of 2021. All input parameter values with upper and lower limits used in the base case analysis and sensitivity analysis are presented in Table 1.

Effectiveness Data: We used quality-of-life scores from an Indian study that used 36 items short-form survey (SF-36) for cured TB patients [27,28]. For parameters pertaining to the quality of life score, lost-to-follow-up, failure, recurrence and, drug-resistant TB patients, we used the scores published from Nigeria [29]. The utility value of well-being was measured on a scale of 0 to 1 which represents death and perfect health, respectively. After the scores had been calculated for cured sensitive TB patients for each domain (including physical functioning, social functioning, role limitation due to physical problems, role limitations due to emotional problems, body pain, general health perception, vitality, and mental health) aggregate scores were calculated and used for the modeling.

Transition Probabilities: The transition probabilities for clinical outcomes of drug-sensitive TB such as cure, lost-to-follow-up, failure, and death for the 4-month regimen were collected from the randomized controlled clinical trial and clinical outcomes for the 6-month regimen were collected from the NTEP reports. The transition probability for ADR of first-line anti-TB drugs was extracted from a prospective multicentric study conducted among TB patients in India. The transition probability for multidrug-resistant TB (MDR-TB) among new cases was used from a published systematic review and meta-analysis [30].

### 2.7. Model Outcome Parameters

The outcomes of the model were expressed in terms of QALYs gained, LYs gained, and overall cost incurred per patient in both 4-month and 6-month regimens. The model compared the incremental cost with incremental QALYs to obtain ICER. The net monetary benefit (NMB) and incremental net monetary benefit (INMB) were calculated.

### 2.8. Willingness to Pay (WTP)

One-time gross domestic product (GDP) per capita (USD 1900) [31] for the year 2020 was used as the WTP threshold and ICERs were used to compare this threshold to determine its cost-effectiveness [32].

### 2.9. Sensitivity Analysis

We randomly generated 1000 input sets by sampling uniformly from the plausible ranges on input parameters. The robustness of the model was tested through sensitivity analysis by varying the input parameters between 20% above or below normal values if the probability exceeded more than one was adjusted. We measured goodness-of-fit as the absolute difference between the target value and the corresponding model output. One-way sensitivity analysis (OWSA) was used to find out the effect of input parameter variations on model outcomes. In the first step, we considered all parameters for OWSA. Further, we used top parameters, which have more influence on the ICER. The uncertainty in outcome variables and their effect on ICER was depicted in the Tornado diagram. Probabilistic Sensitivity Analysis (PSA) using Monte Carlo simulations for 1000 iterations with a 95% confidence interval further was used to validate the model. We evaluated this decision over a 44-year lifetime horizon discounting costs and benefits at 3% per year. The results were presented as a scatter plot and Cost-Effectiveness Acceptability Curve (CEAC).

### 2.10. Model Calibration

For model calibration, we used goodness-of-fit measures to cross-check our results with real-world data. We applied the mortality rate and overall TB treatment cost deviation. The mean Absolute Percentage Deviation (MAPD) test was used to find out the percentage deviation from the observed and estimated values. The following formula was used to calculate the MAPD

$$\mathrm{MAPD}=\frac{\sum_{i=1}^{2}\left|est_{i}-obs_{i}\right|}{obs_{i}}$$

where *est_i_* is the estimated value of the ith endpoint and *obs_i_* is the observed value of the ith endpoint [33].

### 2.11. Study Oversight

This manuscript was reviewed and approved by the manuscript review committee and research integrity committee of ICMR-NIRT, Chennai. Since this modeling was performed with secondary data based on published literature available in the public domain, the study does not require Institutional Ethics Committee Approval. We conducted this study following good reporting practices from published standard guidelines for conducting and reporting an economic evaluation survey (CHEERS) statement (Table S4).

## 3. Results

### 3.1. Base Case Analysis

The base case analysis showed that the total discounted cost incurred for a 4-month and 6-month regimen was USD 14,345,701 and USD 15,039,242, respectively (Table 2). The distribution of various health system costs for the 4-month vs. 6-month regimen include drugs (USD 6,168,386 vs. USD 2,833,792), human resources (USD 2,538,929 vs. USD 3,677,731), ADR hospitalization (USD 1,374,212 vs. 2,675,829) and MDR-TB treatment (USD 428,021 vs. USD 535,491). It was observed that drug cost was higher for the 4-month regimen while cost related to ADR, human resources and MDR-TB treatment was higher for the 6-month regimen. With respect to patient cost for the 4-month vs. 6-month regimen include direct non-medical cost which include food (USD 373,077 vs. USD 540,409), travel (USD 2,150,405 vs. USD 3,114,941) and indirect cost which include loss of income USD 537,117 vs. USD 778,037. Overall patient cost for the 4-month vs. 6-month regimen was USD 3,060,598 vs. USD 4,433,387 respectively. In terms of patient cost, the 4-month regimen was found to be cost saving to the patients.

In terms of effectiveness, the 4-month regimen yielded a higher discounted LY’s (3,152,643 vs. 3,002,584) and QALY’s (2,732,616 vs. 2,601,440) than the 6-month regimen. The 6-month regimen was cost-saving and the incremental discounted cost was USD −693,541 (Table 2). The incremental effectiveness in terms of discounted LYs and QALYs gained for the 4-month was 150,059 and 131,176 respectively. The ICER calculated using discounted LY and QALY was USD −4.62 and USD −5.29, respectively (Table 2). The incremental cost-effectiveness plane plotted indicates that the 4-month regimen is less expensive and more effective when compared with the 6-month regimen (Figure S3). The total NMB estimated from the simulation of the cohort was USD 5162 million and USD 4912 million for the 4-month and 6-month, respectively. The 4-month regimen had a higher INMB of USD 249 million. As per the India TB Report 2021, a total of 1,805,670 TB patients were notified by the NTEP per year, among them, 87.7% were sensitive to TB. If a 4-month regimen was implemented, the estimated NMB was USD 5180 million per year.

### 3.2. Sensitivity Analysis

**Figure 2:**
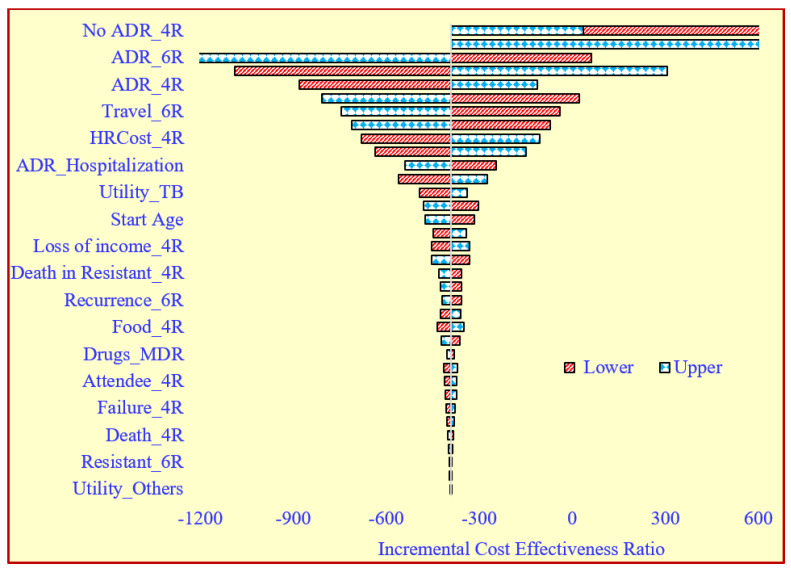
Tornado plot illustrating the one-way sensitivity analysis to see the impact of variation in input parameters on ICER. 4R—Four-month regimen, 6R—Six-month regimen, HR—Human resource, ADR—Adverse Drug Reaction, MDR—Multidrug-resistant.

**Figure 3:**
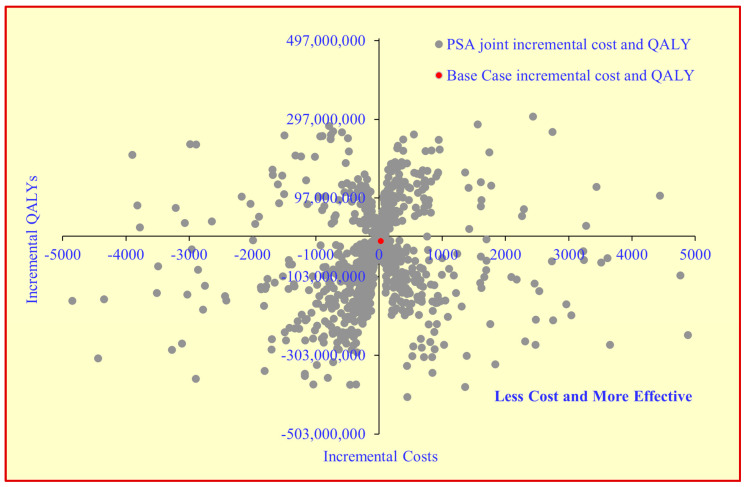
The cost-effectiveness plane for the impact of joint uncertainty of all the input parameters on ICER value. PSA—Probability sensitivity analysis, QALY—Quality adjusted life years.

The OWSA showed that the no ADR 4-month regimen, ADR of the 6-month regimen, ADR of the 4-month regimen, travel for the 6-month regimen, human resource for the 4-month regimen, ADR hospitalization and quality of life (utility) had a higher influence on the ICER value (Figure 2). The PSA highlighted that the joint incremental cost and effectiveness using QALY were less costly and more effective for 67% of the iteration values (Figure 3). The CEAC highlights that the 4-month regimen has a 67% chance of being an economically dominant strategy as compared to the 6-month regimen at different cost-effectiveness threshold values (Figure S4).

### 3.3. Model Calibration

Based on the goodness-of-fit measure the results showed that the predicted cost of the sensitive TB regimen was USD 32 million per 100,000 TB patients. However, the actual cost spent by the programme which included diagnosis, drugs for both sensitive and all-resistant TB, treatment procedures and management costs was USD 62 million per 100,000 TB patients. The other aspects in terms of mortality, the model estimated 2200 per 100,000 TB patients, whereas the actual mortality reported by the programme was 4371 per 100,000 TB patients. The difference between the current model based estimated costs and mortality vs. actual programme reported cost and mortality varied by 48%. Since the current model estimated only for drug sensitive TB patients, whereas the programme reported costs includes both sensitive and all resistance TB patients, the difference was observed.

## 4. Discussion

While the importance of the 4-month regimen is being increasingly recognized globally [34], there is a paucity of research on the economic aspects of this proposed regimen. Efforts to develop a regimen that can shorten the duration of TB treatment have been strongly recommended. With the available clinical efficacy data, we have attempted to evaluate the economic cost and benefits which could be incurred by the introduction of a clinically validated Moxifloxacin-based 4-month regimen. For the first time in India, we assessed the economic impact of using a 4-month regimen for drug-sensitive TB treatment. Our finding highlights that the 4-month regimen was dominant over the 6-month regimen and the total NMB estimated was USD 5180 million per year per cohort of 100,000 drug-sensitive TB patients. The results conclude that the 4-month regimen has a 0.7 probability of being an economically dominant strategy as compared with the current regimen used under NTEP in India.

Our model estimates that introducing this 4-month first-line TB regimen would be dominant in terms of the number of LYs saved and QALYs gained as compared to the present 6-month regimen followed under the NTEP in India. Our evidence on the cost-saving nature of the 4-month regimen is of importance for India and similar high TB burden countries with limited resources to combat TB. While the importance of clinical efficacy and treatment adherence-related benefits of a 4-month regimen have been highlighted in earlier studies our model fulfills the evidence gap concerning the health economic and monetary aspects of a shorter regimen.

TB is often recognized as a disease of poverty and it affects economically disadvantaged populations disproportionately. One of the ‘END TB’ strategies is to reduce the catastrophic cost due to TB-affected families immediately. This goal is challenging as more than half of TB patients have experienced financial difficulties due to the direct and indirect costs incurred by TB care, particularly in high TB burden countries like India and China [35,36]. Our finding underscores that the 4-month regimen is cost-saving for patients. Thus the economic benefits to patients due to the 4-month regimen may address the catastrophic burden by reducing patient costs and may prevent patients from falling into poverty due to TB [37].

Our study findings corroborate the previous model-based study’s findings on the cost-effectiveness of the 4-month regimen. Our study showed the 4-month regimen saved more LYs for a lifetime horizon. Following these consistent results, there is a need to prioritize the implementation of a 4-month regimen at the program level for TB elimination.

The key strength of the present economic evaluation is that it considered a societal perspective to understand the implications of a 4-month regimen for both the patient and provider. Our study findings corroborate an earlier modeling work which reported that the 4-month regimen could reduce the cost to patient and provider. Our findings emphasize the importance of addressing the cost incurred for drugs, travel, and hospitalization for ADR management which were found to be influencing ICER values. This suggests that for large-scale implementation of the 4-month regimen price negotiation of drugs through bulk purchase could be considered. This model holds some limitations. The model parameter for the effectiveness of the 4-month regimen was derived from a clinical trial that could differ from the field conditions, whereas effectiveness for the 6-month regimen was derived from the NTEP report 2020. This difference in terms of effectiveness could be considered a limitation of this model which could be addressed by updating the model with the availability of field data in the future.

## 5. Conclusions

In conclusion, our model results suggest that implementing the 4-month regimen is cost-saving to patients and the health system in India, which may apply to similar resource-limited and high TB burden settings.

## Figures and Tables

**Table 1:** Input parameters used for cost-effectiveness analysis of the 4-month shortened treatment regimen as first-line treatment as compared to the 6-month regimen.

	Input Parameters	Base Case	Lower	Upper	Distribution	Source
Demographic values	Average age of TB patient	32	26	38	Normal	17
	Cohort population	100,000	100,000	100,000	NA	Assumption
	Life expectancy at age 32	44	44	44	NA	18
Mortality	All-cause mortality	0.095	0.008	0.011	Beta	18
Treatment outcome of 4-month regimen	Mortality due to TB	0.003	0.002	0.004	Beta	11
	Failure	0.020	0.016	0.024	Beta	11
	Cure	0.920	0.736	1.000	Beta	11
	Lost-to-follow-up (LTF)	0.040	0.032	0.048	Beta	11
	Adverse drug reaction	0.065	0.052	0.078	Beta	11
Treatment outcome of 6-month regimen	Mortality due to TB	0.041	0.033	0.049	Beta	12
	Failure	0.010	0.008	0.012	Beta	12
	Cure	0.930	0.744	1.000	Beta	13
	Lost-to-follow-up	0.040	0.032	0.048	Beta	12
	Adverse drug reaction	0.130	0.104	0.156	Beta	14
Recurrence	4-month regimen	0.041	0.033	0.049	Beta	11
	6-month regimen	0.069	0.055	0.083	Beta	12
Treatment outcome of LTF	Remain LTF	0.200	0.160	0.240	Beta	14
	Cure	0.630	0.504	0.756	Beta	14
	Failure among LTF	0.107	0.085	0.128	Beta	14
Treatment outcome of failures	Remain failure	0.420	0.336	0.504	Beta	14
	LTF	0.710	0.568	0.852	Beta	14
	Cure	0.420	0.336	0.504	Beta	14
Treatment outcome of recurrence	LTF among recurrence	0.110	0.088	0.132	Beta	14
	Failure	0.950	0.760	1.000	Beta	14
Prevalence of DR-TB		0.290	0.232	0.348	Beta	31
Treatment outcome of DR-TB	Alive	0.890	0.712	1.000	Beta	12
	Death	0.110	0.088	0.132	Beta	12
Quality of life	Utility (cured TB patients)	0.870	0.696	1.000	Beta	28, 29
	Utility (LTF, failure, recurrence, MDR-TB)	0.623	0.498	0.747	Beta	30
Cost for 4-month regimen	Drugs	USD 72	USD 57	USD 86	Gamma	19
	Investigations	USD 5	USD 4	USD 6	Gamma	20
	Hospitalization for ADR	USD 246	USD 197	USD 296	Gamma	22
	Staff	USD 30	USD 24	USD 35	Gamma	21
	Loss of income-patient	USD 6	USD 5	USD 7	Gamma	27
	Food-patient	USD 4	USD 3	USD 5	Gamma	24
	Travel-patient	USD 25	USD 20	USD 30	Gamma	25
	Attendee cost	USD 2	USD 2	USD 3	Gamma	26
Cost for 6-month regimen	Drugs	USD 34	USD 27	USD 41	Gamma	19
	Investigation	USD 5	USD 4	USD 6	Gamma	20
	Hospitalization for ADR	USD 246	USD 197	USD 296	Gamma	22
	Staff	USD 44	USD 35	USD 53	Gamma	21
	Loss of income-patient	USD 9	USD 7	USD 11	Gamma	27
	Food-patient	USD 7	USD 5	USD 8	Gamma	24
	Travel-patient	USD 38	USD 30	USD 45	Gamma	25
	Attendee cost	USD 3	USD 2	USD 4	Gamma	26
Treatment for DR-TB	Drug	USD 274	USD 219	USD 329	Gamma	19
	Investigations	USD 12	USD 10	USD 15	Gamma	20
	Staff	USD 66	USD 53	USD 80	Gamma	22
	Travel-patient & attendee	USD 35	USD 27	USD 40	Gamma	25, 26
	Hospital stay-patient	USD 0.490	USD 0.390	USD 0.580	Gamma	25
	Food-patient	USD 7	USD 6	USD 7	Gamma	24
	Travel-patient	USD 13	USD 10	USD 15	Gamma	25
Willingness to pay threshold	Willingness to pay threshold (GDP per capita) (in Indian Rupees)	USD 1900	-	-	NA	32

NA = Not Applicable; LTF = Lost-to-follow-up; TB = Tuberculosis; ADR = Adverse Drug Reaction; GDP = Gross Domestic Product.

**Table 2:** Incremental cost-effectiveness of the 4-month as compared to the 6-month shortened TB treatment regimen.

	Discounted							
Strategy	Total			Incremental			ICER	
	Cost	Life Years	QALY	Cost	Life Years	QALY	Life Years	QALY
4-month regimen	USD 14,345,701	3,152,643	2,732,616	USD −693,541	150,059	131,176	USD −4.62	USD −5.29
6-month regimen	USD 15,039,242	3,002,584	2,601,440	-	-	-	-	-
	Undiscounted							
4-month regimen	USD 17,047,946	3,545,629	3,073,028	USD −762,033	182,370	158,727	USD −4.18	USD −4.80
6-month regimen	USD 17,809,978	3,363,259	2,914,301	-	-	-	-	-

QALY = Quality Adjusted Life Year; ICER = Incremental Cost Effectiveness Ratio.

## Data Availability

All data generated during this study are included in this published article and its Supplementary Information Files.

## Supplementary Materials

The following supporting information can be downloaded at: https://www.mdpi.com/article/10.3390/tropicalmed7100288/s1, Figure S1: Decision Tree; Figure S2: Markov Model; Figure S3: The cost-effectiveness plane for treatment regimen of smear-positive drug-sensitive pulmonary TB; Figure S4: Cost-Effectiveness Acceptability curve; Table S1: Treatment intervention for adult new smear-positive drug-sensitive pulmonary TB; Table S2: Definition for treatment outcome for adult new smear-positive drug-sensitive pulmonary TB; Table S3: Details of the study population from different studies; Table S4: CHEERS checklist—Items to include when reporting economic evaluations of health interventions; Flie S1: Cost-effectiveness of shorter four-month tuberculosis treatment regimen in India.
