# Supplementary material for: Cost Effectiveness of a Shorter Moxifloxacin Based Regimen for Treating Drug Sensitive Tuberculosis in India
Source: Trop Med Infect Dis. 2022 Oct 8;7(10):288. doi: 10.3390/tropicalmed7100288 (PMC9607110; doi:10.3390/tropicalmed7100288)
Supplement: Supplementary file 1 [file tropicalmed-07-00288-s001.zip › tropicalmed-1943247-supplementary.pdf]

Supplementary Appendix

Table S1. Treatment intervention for adult new smear-positive drug-sensitive pulmonary TB.

Strategies	Drugs	Regimen	Duration	Population
Current strategy	Isoniazid (H)<br>Rifampicin (R)<br>Pyrazinamide (Z)<br>Ethambutol (E)	$2HRZE_7/4RHE_7$	6-months	Adult new smear-positive drug-sensitive pulmonary TB
Comparator	Moxifloxacin (M)<br>Isoniazid (H)<br>Rifampicin (R)<br>Pyrazinamide (Z)<br>Ethambutol (E)	$2RHZEM_7/2RHM_7$	4-months	Adult new smear-positive drug-sensitive pulmonary TB

Table S2. Definition for treatment outcome for adult new smear-positive drug-sensitive pulmonary TB.

Treatment outcomes	Definition
**Cure**	The cure is a pulmonary TB patient with bacteriologically confirmed TB at the beginning of treatment who was smear or culture-negative in the last month of treatment and on at least one previous occasion
**Lost to follow-up**	Lost to follow-up are those TB patients whose treatment was interrupted for two consecutive months or more
**Treatment failure**	Treatment failure is considered when a patient is sputum smear or sputum culture-positive in the second and last month of treatment or later after the initiation of anti TB treatment
**Death**	Death is a TB patient who dies for any reason before starting or during treatment
**Recurrent TB**	Recurrent TB in patients who have previously been treated for TB were declared cured and are now diagnosed with a recurrent episode of TB (either a true relapse due to reactivation of the disease or a new episode of TB).

Table S3. Details of the study population from different studies.

Patient Characteristics	No (%)
Total	1329 *(100%)*
Gender	
Male	1000 (75)
Female	329 (25)
Age (Years)	
<35	641 (48)
≥35	688 (52)

Source: Velayutham B, Jawahar MS, Nair D, et al. 4-month moxifloxacin containing regimens in the treatment of patients with sputum-positive pulmonary tuberculosis in South India - a randomised clinical trial. *Trop Med Int Health*. (2020). 25(4): 483-495.

Patient Characteristics	No (%)
Total	*227 (100)*
Gender	
Male	133 (57)
Female	94 (41)
Age (Years)	
0-9	3 (1)
10-19	46 (20)
20-60	164 (72)
>60	14 (6)

Source: Sharma P, Verma M, Bhilwar M, et al. Epidemiological profile of tuberculosis patients in Delhi, India: A retrospective data analysis from the directly observed treatment short-course (DOTS) center. *J Family Med Prim Care*. (2019). 8(10): 3388-3392.

Patient Characteristics	Age (Year)		
	**Men Mean ± (SD)**	**Women Mean ± (SD**	**Average Mean ± (SD**
Center 1	29.29 ± (15.195)	31.89 ± (16.193)	30.50 ± (15.700)
Center 2	31.09 ± (13.552)	23.00 ± (11.205)	27.90 ± (13.209)
Center 3	34.00 ± (16.504)	23.14 ± (10.518	28.38 ± (14.665)
Center 4	28.45 ± (14.873)	25.00 ± (9.808)	26.34 ± (12.973)
Center 5	33.40 ± (15.415)	34.62 ± (15.538)	33.88 ± (15.231)
Center 6	29.85 ± (8.732)	39.14 ± (25.354)	32.26 ± (14.875)
All Centers	30.60 ± (14.435)	27.57 ± (14.485)	29.30 ± (14.514)

Source: Imam F, Sharma M, Khayyam KU, et al. Adverse drug reaction prevalence and mechanisms of action of first-line anti-tubercular drugs. *Saudi Pharm J*. (2020). 28(3):316-324.

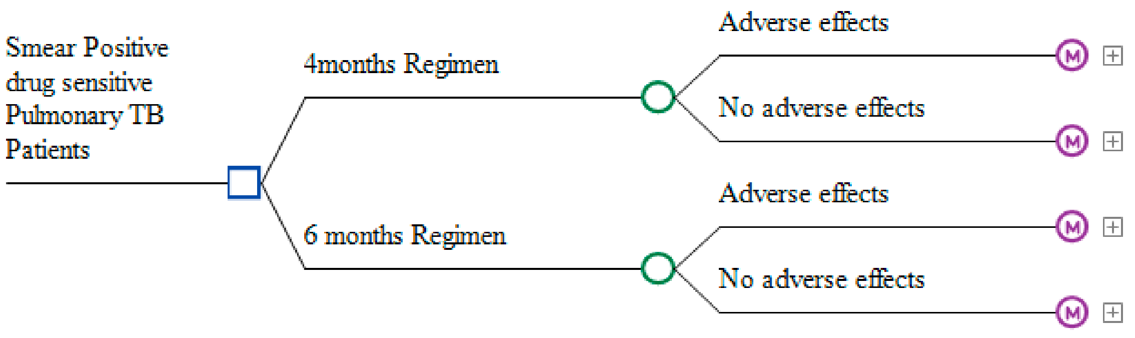

Figure S1. Decision Tree.

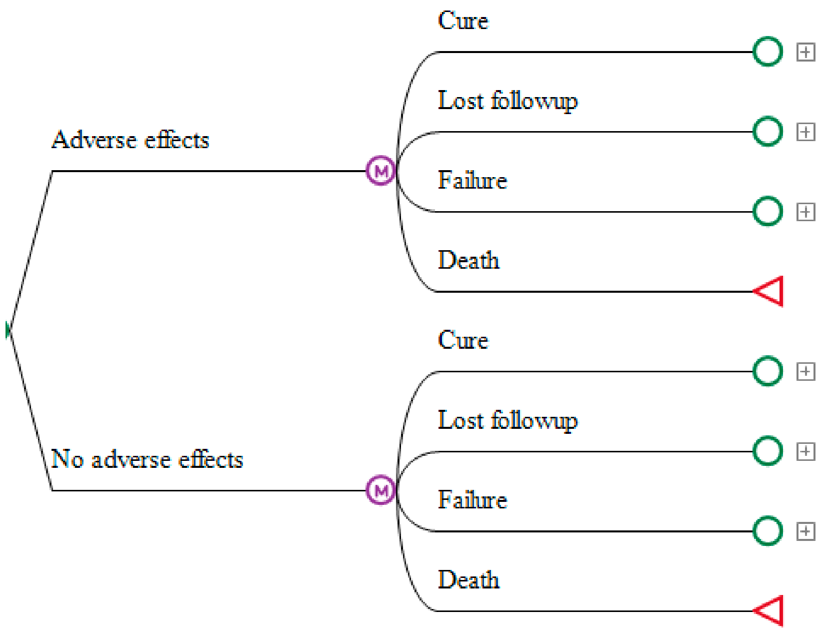

Figure S2. Markov Model.

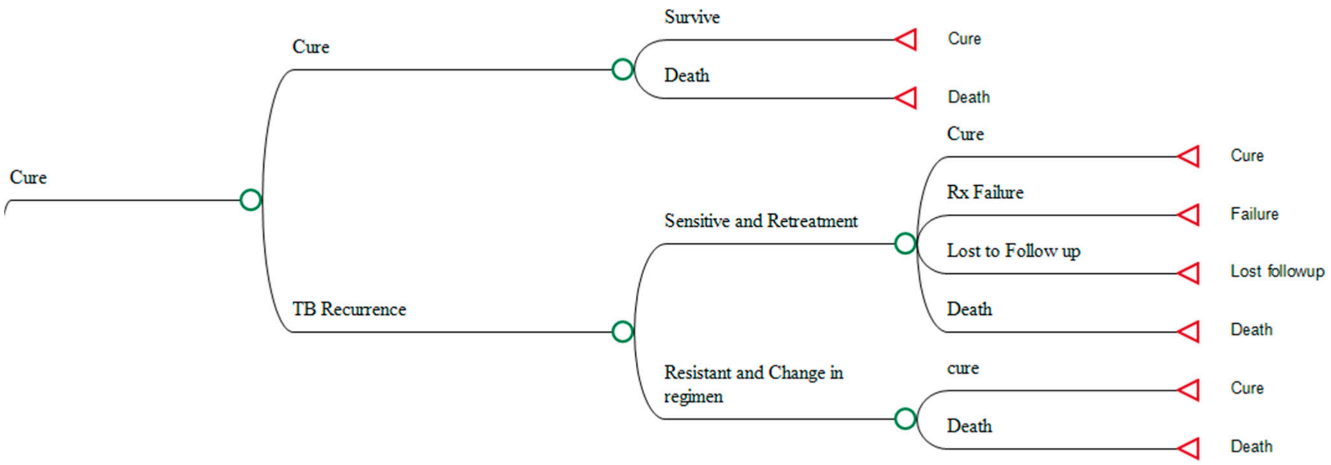

Figure S2. a. Cure Node.

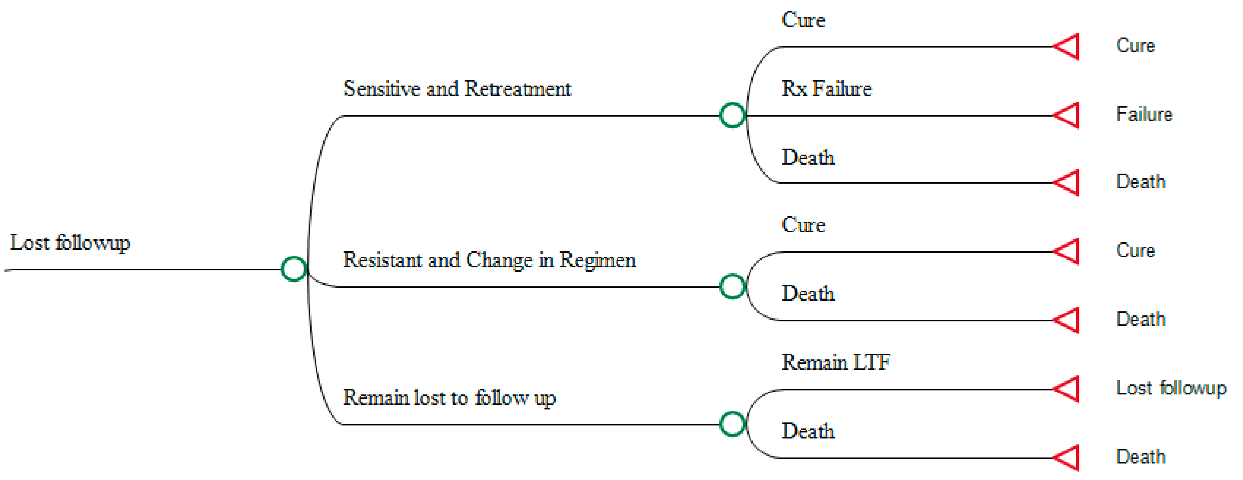

Figure S2. b. Lost-to-follow-up.

Rx=Treatment

LTF=Loss to Follow-up

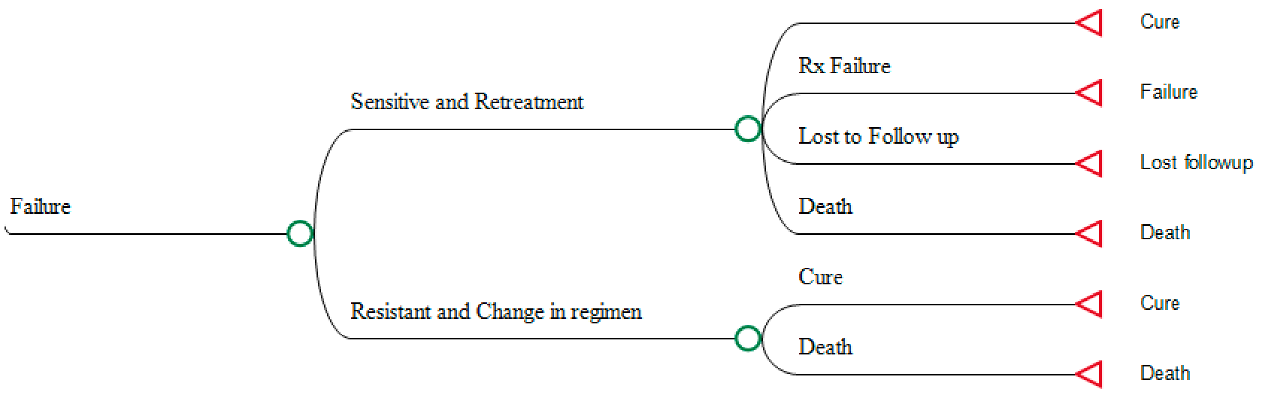

Figure S2. c. Failure node.

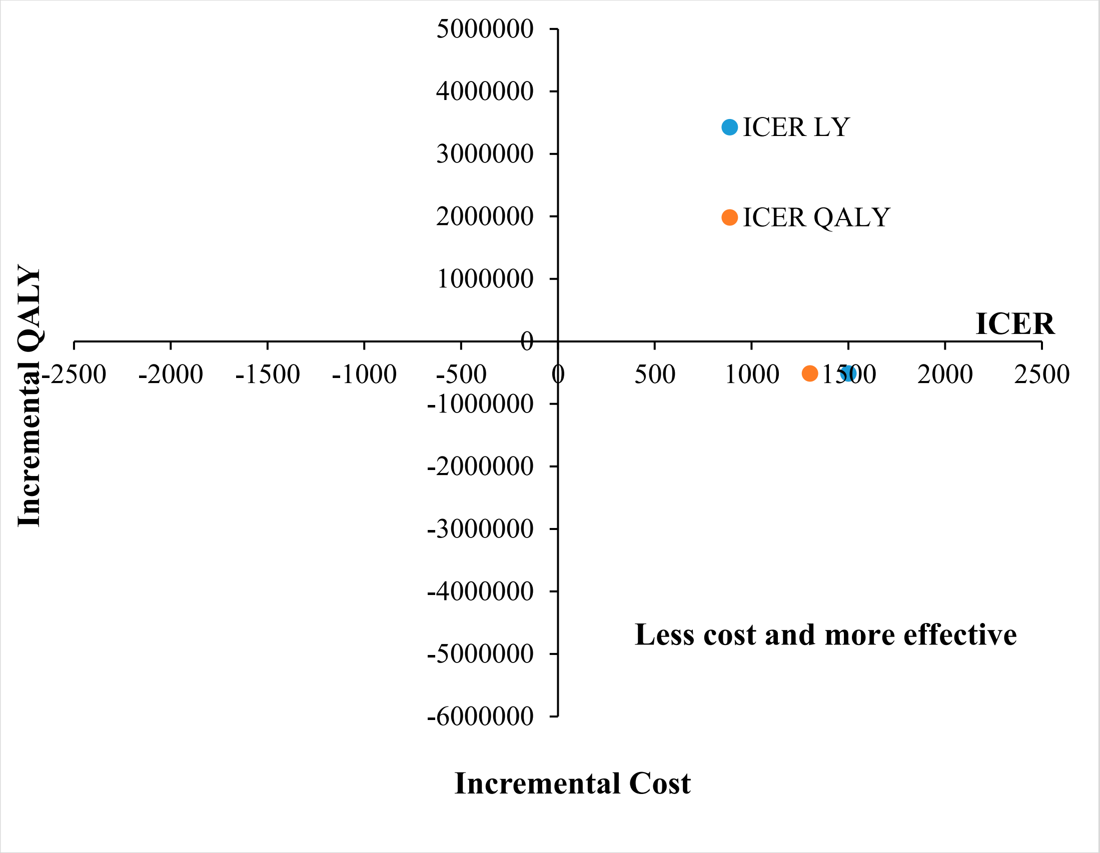

Figure S3. The cost-effectiveness plane for treatment regimen of smear-positive drug-sensitive pulmonary TB.

Incremental Cost Effectiveness Ratio
LY=Life Years
QALY=Quality Adjusted Life Years

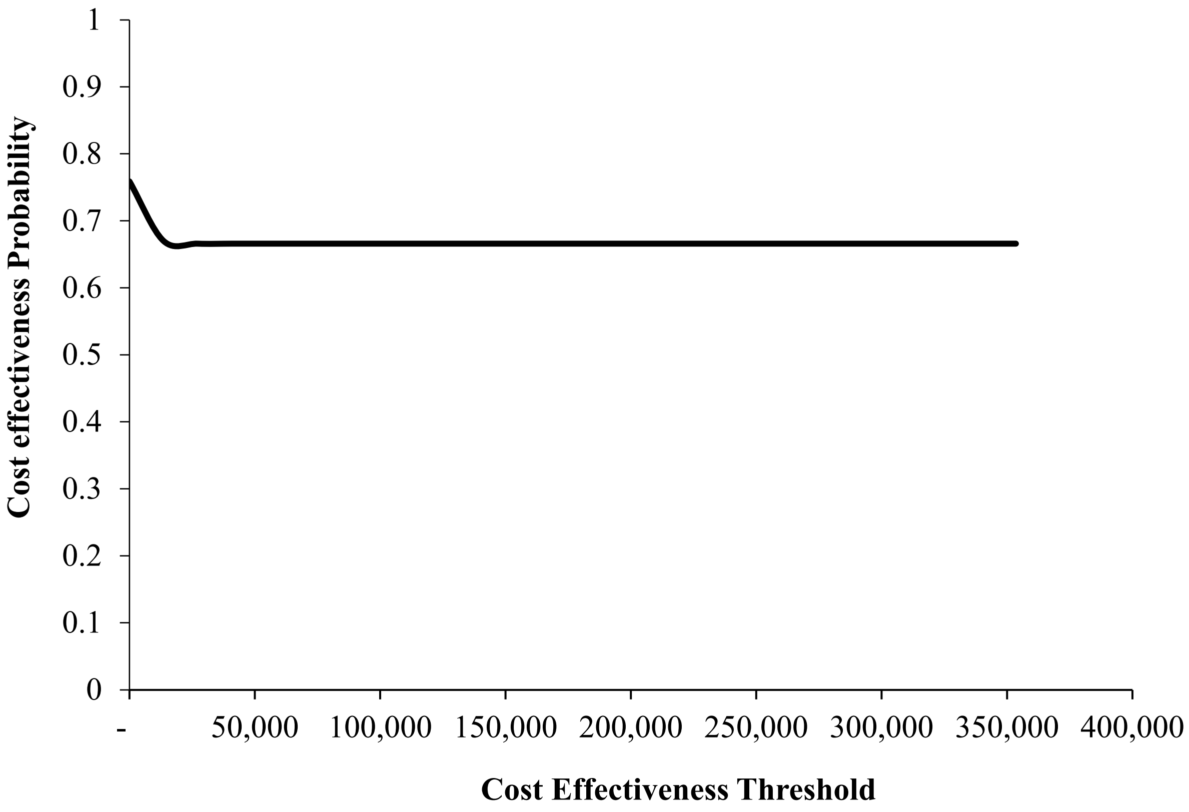

Figure S4. Cost-Effectiveness Acceptability curve.

Table S4. CHEERS checklist—Items to include when reporting economic evaluations of health interventions.

Section/item	Item No	Recommendation	Reported on page No/ line No
**Title and abstract**			
Title	1	Identify the study as an economic evaluation or use more specific terms such as "cost-effectiveness analysis", and describe the interventions compared.	Page 1
Abstract	2	Provide a structured summary of objectives, perspective, setting, methods (including study design and inputs), results (including base case and uncertainty analyses), and conclusions.	Page 2
**Introduction**			
Background and objectives	3	Provide an explicit statement of the broader context for the study.	Page 2
		Present the study question and its relevance for health policy or practice decisions.	Page 2
**Methods**			
Target population and subgroups	4	Describe characteristics of the base case population and subgroups analysed, including why they were chosen.	Page 2
Setting and location	5	State relevant aspects of the system(s) in which the decision(s) need(s) to be made.	Page 2
Study perspective	6	Describe the perspective of the study and relate this to the costs being evaluated.	Page 2
Comparators	7	Describe the interventions or strategies being compared and state why they were chosen.	Page 2
Time horizon	8	State the time horizon(s) over which costs and consequences are being evaluated and say why appropriate.	Page 2
Discount rate	9	Report the choice of discount rate(s) used for costs and outcomes and say why appropriate.	Page 3
Choice of health outcomes	10	Describe what outcomes were used as the measure(s) of benefit in the evaluation and their relevance for the type of analysis performed.	Page 3
Measurement of effectiveness	11a	*Single study-based estimates:* Describe fully the design features of the single effectiveness study and why the single study was a sufficient source of clinical effectiveness data.	Not Applicable
	11b	*Synthesis-based estimates*: Describe fully the methods used for identification of included studies and synthesis of clinical effectiveness data.	Page 4
Measurement and valuation of preference based outcomes	12	If applicable, describe the population and methods used to elicit preferences for outcomes.	Not Applicable
Estimating resources and costs	13a	*Single study-based economic evaluation:* Describe approaches used to estimate resource use associated with the alternative interventions. Describe primary or secondary research methods for valuing each resource item in terms of its unit cost. Describe any adjustments made to approximate to opportunity costs.	Not Applicable
	13b	*Model-based economic evaluation:* Describe approaches and data sources used to estimate resource use associated with model health states. Describe primary or secondary research methods for valuing each resource item in terms of its unit cost. Describe any adjustments made to approximate to opportunity costs.	Page 4
Currency, price date, and conversion	14	Report the dates of the estimated resource quantities and unit costs. Describe methods for adjusting estimated unit costs to the year of reported costs if necessary. Describe methods for converting costs into a common currency base and the exchange rate.	Page 5
Choice of model	15	Describe and give reasons for the specific type of decision-analytical model used. Providing a figure to show model structure is strongly recommended.	Page 3&4
Assumptions	16	Describe all structural or other assumptions underpinning the decision-analytical model.	Page 3
Analytical methods	17	Describe all analytical methods supporting the evaluation. This could include methods for dealing with skewed, missing, or censored data; extrapolation methods; methods for pooling data; approaches to validate or make adjustments (such as half cycle	Page 6&7

Section/item	Item No	Recommendation	Reported on page No/ line No
		corrections) to a model; and methods for handling population heterogeneity and uncertainty.	
		**Results**	
Study parameters	18	Report the values, ranges, references, and, if used, probability distributions for all parameters. Report reasons or sources for distributions used to represent uncertainty where appropriate. Providing a table to show the input values is strongly recommended.	Page 7&8
Incremental costs and outcomes	19	For each intervention, report mean values for the main categories of estimated costs and outcomes of interest, as well as mean differences between the comparator groups. If applicable, report incremental cost-effectiveness ratios.	Page 8
Characterising uncertainty	20a	*Single study-based economic evaluation:* Describe the effects of sampling uncertainty for the estimated incremental cost and incremental effectiveness parameters, together with the impact of methodological assumptions (such as discount rate, study perspective).	Not Applicable
	20b	*Model-based economic evaluation:* Describe the effects on the results of uncertainty for all input parameters, and uncertainty related to the structure of the model and assumptions.	Page 8,9 &10
Characterising heterogeneity	21	If applicable, report differences in costs, outcomes, or cost-effectiveness that can be explained by variations between subgroups of patients with different baseline characteristics or other observed variability in effects that are not reducible by more information.	Not Applicable
Study findings, limitations, generalisability, and current knowledge	22	Summarise key study findings and describe how they support the conclusions reached. Discuss limitations and the generalisability of the findings and how the findings fit with current knowledge.	Page 11
Source of funding	23	Describe how the study was funded and the role of the funder in the identification, design, conduct, and reporting of the analysis. Describe other non-monetary sources of support.	Page 12
Conflicts of interest	24	Describe any potential for conflict of interest of study contributors in accordance with journal policy. In the absence of a journal policy, we recommend authors comply with International Committee of Medical Journal Editors' recommendations.	Page 12

Flie S1

Cost-effectiveness of shorter four-month tuberculosis treatment regimen in India

TECHNICAL NOTE

Definitions

Smear positive pulmonary TB patients

Newly diagnosed adult sputum positive pulmonary TB patients with no previous history of TB. Excluding those with extra-pulmonary TB, hepatic or renal disease and HIV.

TB Recurrence

TB relapse in this study is a patient who has become (and remained) culture-negative while receiving therapy but after completion of therapy becomes culture-positive again or has clinical or radiographic deterioration that is consistent with active tuberculosis.

Treatment failure

Treatment failure of TB, in this study is defined as a patient who is sputum smear or sputum culture-positive in the second and last month of treatment or later after the initiation of anti TB treatment

Lost-to-follow-up (LTF)

In this study, we have considered all smear-positive TB patients whose treatment was interrupted for two consecutive months or more as lost to follow up.

Adverse Drug Reaction (ADR)

Those patients who developed adverse drug reaction attributable to drugs in the treatment regimen. The ADR in this study includes only cases of moderate and severe as it requires hospitalisation and patients with mild ADR were excluded as they required only symptomatic treatment.

Health system cost

Health system cost refers to the cost incurred by the provider. The health system in this study is a public health facility that provides medical services at a subsidised rate or free of cost. Thus all medical cost was assumed to be incurred only by the public health facility. This includes Human Resource, Infrastructure, Equipment, Investigation, Treatment and Medication cost.

Patient cost

This includes travel cost, food cost, other out-of-pocket expenditure for availing TB treatment services and indirect cost (productivity loss)

Indirect costs

Indirect costs refer to the loss of income resulting from work absenteeism, interruption of normal or preferred activities of the patient and household members. The average loss of wage per patient per treatment course is considered.

Time Horizon

When designing comparative outcomes or cost-effectiveness analysis, the time horizon defining the duration of time for outcomes assessment considered. The time horizon must be long enough to capture the intended and unintended benefits and harms of the intervention. This study considers a lifetime horizon to capture all cost and effectiveness.

Decision Tree

A decision tree is the most powerful and popular tool for classification and prediction. A Decision tree is a flowchart like a tree structure, where each internal node denotes a test on an attribute, each branch represents an outcome of the test, and each leaf node (terminal node) holds a class label.

Cost-effectiveness analysis

Cost-effectiveness analysis (CEA) is a way to examine both the costs and health outcomes of one or more interventions. It compares an intervention to another intervention (or the status quo) by estimating how much it costs to gain a unit of a health outcome like a life-year gained or death prevented.

Incremental cost-effectiveness ratio

The incremental cost-effectiveness ratio (ICER) between the current 6 month TB treatment strategy with the new intervention of 4 month TB Treatment strategy. The difference between the strategies was calculated and compared with the difference in the number of QALYs gained.

ICER = Cost of the proposed TB treatment strategy (4 months) – Cost of the current TB
Treatment strategy (6 month)

QALY of the proposed TB treatment strategy (4 months) – QALY of the current TB Treatment strategy (6 months)

Net Monetary Benefit

Net Monetary benefit (NMB) is a summary statistic that represents the value of an intervention in monetary terms considering a willingness to pay threshold for a unit of benefit. This study considers per capita GDP as the willingness to pay threshold

NMB= (QALY * Willingness to pay Threshold) – Cost

Incremental NMB (INMB) measures the difference in NMB between alternative interventions, a positive incremental NMB indicating that the intervention is cost-effective compared with the alternative at the given willingness-to-pay threshold.

INMB= NMB (Proposed Strategy) – NMB (Current Strategy)

Quality Adjusted Life Year

The Quality-Adjusted Life Year (QALY) is a standardized measure of disease burden which combines both survival and health-related quality of life into a single index. The QALY is primarily used in cost-effectiveness analyses to guide decisions regarding the distribution of limited health care resources among competing health programs or interventions for a population of interest but has also been used to aid decisions regarding clinical management and individual patient care.

QALY= Utility * Expected Life years

One-way sensitivity analysis

Univariate/one-way sensitivity analysis (OWSA) is to assess the impact that changes in a certain input (parameter) will have on the output results of an economic evaluation. This will help to assess the robustness of the result to that parameter. It is helpful for decision-makers to have insights into the relationship between specific input parameters and the model outputs.

Probabilistic sensitivity analysis

Probabilistic sensitivity analysis (PSA) demonstrates the parameter uncertainty in a decision problem. The technique involves sampling parameters from their respective distributions (rather than simply using mean/median parameter values). This technique used in economic modelling allows the modeller to quantify the level of confidence in the output of the analysis, concerning uncertainty in the model inputs.

Cost-effectiveness threshold

The cost-effectiveness threshold is the ceiling ICER beyond which interventions are no longer considered cost-effective, reflecting the maximum value decision-makers attach to health benefits. Three general approaches have been used to provide clear guidance to policymakers: (i) thresholds based on per capita national incomes; (ii) benchmark interventions and (iii) league tables. In recent years, the most common approach has involved the use of thresholds based on GDP. Under this approach which has been promoted by the World Health Organization's Choosing cost-effective interventions (WHO-CHOICE) an intervention that costs less than three times the national annual GDP per capita is considered cost-effective, whereas one that costs less than once the national annual GDP per capita is considered highly cost-effective. We followed GDP as the threshold value.

Calculations

Discount factor calculation

The discount factor is multiplied with life years (LY), quality-adjusted life years (QALY) and cost to obtain discounted LY, QALY and cost.

$$\text{Discount factor} = \frac{1}{1*(1+\text{Discount rate})^{\text{time}}}$$

Discounted Life Years (LY) = LY * Discount factor

Discounted Quality-adjusted life Years (QALY) = QALY * Discount factor

Discounted Cost = Cost * Discount factor

Cost estimation

Costs were obtained from published reports and literature. The staff cost, food cost, travel cost, cost for productivity loss and escort cost for a standard six-month regimen with an intermittent hospital visit for three days a week was available and hence it was converted to per-visit cost. Considering two visits per month in both the regimen per visit cost was used to calculate the cost for both six months and four months treatment regimen. Average hospitalisation cost per patient due to Adverse Drug Reaction was available and was considered similar in both the arm assuming that it is similar in both treatment regimen. The cost available in US$ was converted using the exchange rate from OECD and then the cost for 2020 was adjusted using inflation rate by using the formula

Cost for 2020 = cost in particular year + (cost in particular year*Inflation rate for 2020)

MDR cost

Currently, a shorter regimen of 4 months intensive phase followed by 5 months continuous phase is being advised for MDR treatment. The drugs and dosage for each phase were collected from the TB India Report. The distribution of weight range in MDR-TB was obtained from published literature[19]. MDR drug cost for each unit was collected from National Pharmaceutical Pricing Authority (NPPA), Government of India and the total cost by dosage and duration of treatment was calculated across the weight range.

Prevalence of Resistant TB

The pooled prevalence of resistant TB cases was re-calculated from a systematic review conducted in 2019 through meta-analysis including literature collected only from 2011 to 2016 by using R software and was assumed to be the same for both 4 months and 6-month regimen.

Human Resources

The proportion of time spent for TB services by different health personnel ranges from 100% for personnel working in the district TB centre to <10% for health visitors working in the field. This was taken from the previous studies reported form our centre.
